# Metabolic Health—The Role of Adipo-Myokines

**DOI:** 10.3390/ijms20246159

**Published:** 2019-12-06

**Authors:** Christine Graf, Nina Ferrari

**Affiliations:** 1 Department for physical activity in public health, Institute of Movement and Neurosciences, Am Sportpark Müngersdorf 6, German Sport University Cologne, 50933 Cologne, Germany; 2 Cologne Center for Prevention in Childhood and Youth/Heart Center Cologne, University Hospital of Cologne, Kerpener Str. 62, 50937 Cologne, Germany; nina.ferrari@uk-koeln.de

**Keywords:** adipokine, myokine, fitness, metabolically healthy obese, early-life programming

## Abstract

Obesity is now a worldwide epidemic. In recent years, different phenotypes of obesity, ranging from metabolically healthy normal weight to metabolically unhealthy obese, were described. Although there is no standardized definition for these phenotypes or for metabolic health, the influence of lifestyle and early-life factors is undisputed. In this context, the ratio of muscle-to-fat tissue seems to play a crucial role. Both adipose tissue and skeletal muscle are highly heterogeneous endocrine organs secreting several hormones, with myokines and adipokines being involved in local autocrine/paracrine interactions and crosstalk with other tissues. Some of these endocrine factors are secreted by both tissues and are, therefore, termed adipo-myokines. High (cardiorespiratory) fitness as a surrogate parameter for an active lifestyle is epidemiologically linked to “better” metabolic health, even in the obese; this may be partly due to the role of adipo-myokines and the crosstalk between adipose and muscle tissue. Therefore, it is essential to consider (cardiovascular) fitness in the definition of metabolically healthy obese/metabolic health and to perform longitudinal studies in this regard. A better understanding of both the (early-life) lifestyle factors and the underlying mechanisms that mediate different phenotypes is necessary for the tailored prevention and personalized treatment of obesity.

## 1. Introduction

The overall prevalence of obesity increased dramatically over the last few decades [1,2,3]. The World Health Organization (WHO) reported that more than 1.9 billion adults around the world are overweight, and nearly one-third of the population is obese [4].

From an epidemiological perspective, obesity is linked to so-called non-communicable diseases (NCDs), a set of diseases of long duration and slow progression, including cardiovascular diseases, diabetes mellitus type 2, respiratory diseases, and certain types of cancer [5]. These NCDs kill 41 million people each year, equivalent to 71% of all deaths globally [6]; 1.6 million deaths annually can be attributed to insufficient physical activity [7].

In general, obesity is still defined on the basis of body mass index (BMI), and BMI in itself is generally accepted as a strong predictor of overall early mortality [3]. The increased health risk is particularly linked to high amounts of white and/or visceral fat because of its endocrine activities. Visceral adipose tissue produces more than 600 so-called adipokines that regulate not only metabolic processes such as insulin secretion, hunger and satiety, and energy balance, but inflammatory processes as well [8]. An increasing accumulation of visceral fat in terms of chronic overfeeding results in dysfunctional adipose tissue with excessive adipokine secretion and an altered secretion profile characterized by increased leptin, interleukin (IL)-6, and tumor necrosis factor (TNF)-α levels, as well as increased oxidative stress and a reduction in adiponectin [9], leading to chronic low-grade inflammation.

Whereas physical activity/exercise can be protective against these pathological conditions [10], physical inactivity enhances the inflammatory mechanisms described above [11]. This might be explained by the specific function of skeletal muscle mass. Skeletal muscle is the largest organ in the body; its energy production and consumption are fundamental to metabolic control. Currently, skeletal muscle is also identified as an endocrine organ secreting hundreds of so-called myokines such as myostatin, IL-4, IL-6, IL-7, IL-15, myonectin, follistatin-like 1 (FSTL1), leukemia inhibitory factor, and/or irisin [12,13]. Not only do these myokines act locally in the muscle in an autocrine/paracrine manner, but they are also released into the bloodstream as endocrine factors to regulate physiological processes in other tissues. The release of myokines from contracting muscle is assumed to be at least partly responsible for the health-promoting effects of physical activity that protects against major chronic, low-grade inflammatory diseases like type 2 diabetes, insulin resistance, metabolic syndrome, and many others.

**Figure 1:**
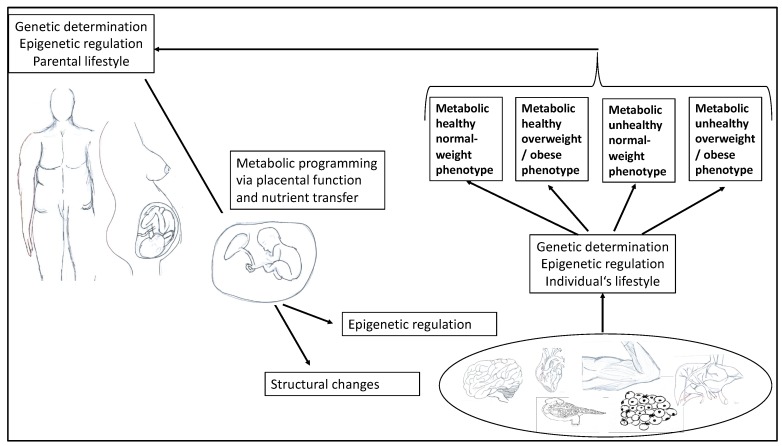
Illustration of the interaction of parental factors on offspring in pregnancy, affecting epigenetic regulation and different organ systems in the development of different obesity phenotypes in terms of metabolic health.

In 2013, Raschke and Eckel [14] described the interplay between muscle and adipose tissue as a two-edged sword. They pointed out that certain cytokines are released by both skeletal muscle and adipose tissue, exhibiting a bioactive effect. The authors suggested calling them adipo-myokines because they are both mediators of exercise and mediators of inflammation. For this reason, as well as for the development of tailored prevention and treatment, it is important to better understand the molecular mechanisms and potential influencing factors that predispose obese (or inactive) individuals to the development of metabolic diseases. Increasing evidence also suggests that early-life exposure to a range of environmental factors, including parental BMI and lifestyle (e.g., maternal nutrition), plays a critical role in defining an offspring’s metabolic health. According to the Developmental Origins of Health and Disease hypothesis, environmental exposures during critical periods—such as the preconception, fetal, and early infant phases of life—can influence development and have a persistent impact on metabolic health and gene expression, thereby influencing offspring phenotype and disease risk in later life (Figure 1) [15,16].

The particular aim of this article is to explore the extent to which biomolecular findings can substantiate the concept of metabolic health and to investigate the role of physical activity/exercise, using cardiorespiratory fitness as a surrogate marker considering early-life factors.

## 2. Definition and Epidemiological Findings

A striking limitation of BMI is shown by the different phenotypes of obesity. In the early 1980s, Ruderman et al. [17] described the manifestation of cardiometabolic risk factors in non-obese subjects, termed “Metabolically Obese, Normal Weight” (MONW); while they are characterized as having hyperinsulinemia, hyperglycemia, insulin resistance, impaired glucose tolerance, hypercholesterolemia, and hypertriglyceridemia, they have normal adipocyte volume and BMI. On the other hand, obese individuals who showed no health risk factors were also described [18]. This phenomenon—called benign obesity or MHO (metabolically healthy obese)—is characterized by a high BMI or high amount of fat mass in the absence of insulin resistance, increased blood lipids, high blood pressure, or inflammatory dysregulation in contrast to metabolically unhealthy obese (MUO). Discussions about whether the manifestation of MHO is clinically relevant, or whether it might be a form of “honeymoon obesity” (alluding to a temporary absence of metabolic illness), are still ongoing. Nonetheless, it is becoming increasingly clear that a distinction between the various forms of obesity is important given the difference in therapeutic approaches [19]. It is likely that fat distribution and/or body composition play a central role due to different health risks.

To date, there is no uniform classification for this phenotype, while up to 30 different definitions were proposed in several studies (summarized in Reference [20]). Depending on which definition is used, information about the prevalence of MHO varies in the literature [21]. The prevalence of MHO is 3% to 32% in men and 11% to 43% in women using BMI, 6% to 37% in men and 12% to 58% in women using abdominal obesity, and 6% to 43% in men and 12% to 56% in women using body fat as the defining parameter. A similar inconsistency in the literature is found regarding the actual manifestation of the condition. As such, some authors do not recognize the phenomenon as a condition itself but suggest that it is a temporary state which will sooner or later result in a pathological condition [22]. In a meta-analysis, Kramer et al. [23] showed that MHO individuals had an increased risk for cardiovascular events compared with metabolically healthy normal-weight (MHNW) individuals when only studies with 10 or more years of follow-up were considered (+24%). In contrast, all metabolically unhealthy groups had a similar risk: normal weight (risk ratio (RR), 3.14), overweight (RR, 2.70), and obese (RR, 2.65).

Approximately 30% to 50% of MHO individuals seem to convert to the MUO phenotype after four to 20 years of follow-up (summarized in Reference [20]). A major factor in this transition seems to be the development of insulin resistance, which is enhanced by a high BMI, weight gain, older age, and a poor lifestyle index (including physical inactivity).

These controversies underline the need for specific, individualized treatments based on individual diagnostic findings and risk. They further highlight the importance of promoting a healthy, active lifestyle. Moreover, additional factors should be included in health risk assessments. Smith et al. [20] suggest basing the risk on the absence of cardiometabolic diseases and on the cardiometabolic profile, including normal blood lipid, blood pressure, and blood glucose levels, as well as intrahepatic fat content, as advanced criteria. From our point of view, however, body composition and cardiorespiratory fitness would be missing as protective factors in this list of criteria, at least of the advanced type. Similarly, Ruderman et al. [17] noted that physical activity should be added as a therapeutic agent, with maximal oxygen uptake added as a criterion, in reference to MONW. However, Velho et al. [21] demonstrated in the CoLaus Study, a cross-sectional investigation aimed at assessing the prevalence and deciphering the molecular determinants of cardiovascular risk factors in the Caucasian population of Lausanne, that MHO individuals were more physically active. For this reason, Stefan et al. [24] suggested defining MHO using the following six parameters: waist circumference, insulin resistance, blood sugar levels, blood pressure, cholesterol levels, and physical fitness.

## 3. Physical Activity, Cardiorespiratory Fitness, and/or Sedentary Behavior and Its Relation to MHO

In general, physical activity is defined as any bodily movement that results in energy expenditure [25]. Exercise is a subset of physical activity that is planned, structured, and repetitive, and that has, as a final or an intermediate objective, the improvement or maintenance of physical fitness. The “dose” is expressed as energy expenditure, and the intensity is expressed as the rate of energy consumption in selected activities, usually expressed as a percentage of VO_2_ max engaged during exercise (or relative to individual body weight) or metabolic units (METs). Activities with <1.5 METs are classified as sedentary or inactivity [26]. The current physical activity recommendations for adults can be found in Table 1.

Even though there is a large consensus about the recommended levels of physical activity, the broad range of methods used to measure such activity makes the final decision-making process complex. Studies may use subjective or objective measurements, test endurance, strength, or co-ordination, and different intensities, volumes, and training methods like interval or continuous training in their intervention. Hence, testing cardiorespiratory fitness levels as a surrogate marker may still be the most sensible option. Many studies showed that well-trained individuals have a better prognosis with regard to NCDs than those with lower fitness levels [28,29]. In the concept of fitness instead of fatness, this would also concern overweight and obese individuals [30]. There are, however, only a few studies investigating these connections with MHOs, and most of them are cross-sectional analyses [31]. Between 2010 and 2013, the Maastricht study investigated the relationship between MHO, MUO, metabolically healthy non-obese (MHNO), and metabolically unhealthy non-obese (MUNO) groups, as well as physical activity and sedentary behavior in 2449 men and women aged 40–70 years [31]. Based on accelerometry data, the MHO group was more active (ca. 15 min) and less sedentary (ca. 30 min) than the MUO group. Furthermore, Camhi et al. [32] analyzed National Health and Nutrition Examination Survey (NHANES) data from obese adolescents and adults from the 2003–2005 cohort. The adult MHO group was 85% more likely to engage in active transportation and nearly three times more likely to be involved in light-intensity, usual daily activity versus sitting (self-reported). A higher level of moderate physical activity was also associated with the MHO group.

In a systematic review and meta-analysis, Ortega et al. [33] explored the differences between physical activity, sedentary behavior, and cardiorespiratory fitness between MHO and MUO individuals, as well as the prognosis of all-cause mortality and cardiovascular disease (CVD) mortality/morbidity in MHO individuals only. The analysis of 67 cross-sectional studies showed that MHO individuals were more active and less sedentary, and they had a higher level of cardiorespiratory fitness. However, cardiorespiratory fitness was only measured in 19 studies, and the difference in VO_2_ peak or VO_2_ max was usually not more than 1–2 mL/kg body weight. Only one study—the Aerobics Center Longitudinal Study—examined the role of cardiorespiratory fitness in the prognosis of all-cause mortality and cardiovascular disease mortality/morbidity, collecting the data of 43,269 participants. This study explained the differences in the risk of mortality and morbidity between MHO and MHNW subjects through the differences found in cardiorespiratory fitness between both groups. The MHO group had a 30% to 50% lower risk of all-cause mortality, cardiovascular disease, and cancer mortality than the MUO group; no significant differences were observed between the MHO and MHNW groups [34]. The median follow-up period for mortality was 14.3 years, and it was7.9 years for non-fatal CVD incidence. In another longitudinal study, Pigłowska et al. [35] demonstrated in 101 men that both fat and muscle mass components are important predictors of an individual’s metabolic profile. Maintaining regular, high physical activity levels, and a metabolically healthy status throughout young and middle adulthood may have a beneficial influence on body composition parameters and may prevent the age-related decrease of fat-free mass and endothelial dysfunction, defined according to the National Cholesterol Education Program Adult Treatment Panel III (NCEP ATP III) guidelines. Moreover, in a six-year-long cohort study of over 200,000 Taiwanese participants, Martinez-Gomez et al. [36] showed that physical activity can cause a shift in status from MUO to MHO.

## 4. Myokines, Adipokines, and Adipo-Myokines

The regulation of metabolic health in obese individuals may be mediated by the effects that an active or inactive/sedentary lifestyle have on individual endocrine and inflammatory responses. Myokines, adipokines, and particularly the so-called adipo-myokines are at the crosstalk between muscle and fat tissue. Raschke and Eckel [14], as well as Görgens et al. [37], defined IL-6, TNF-α, visfatin, myostatin, FSTL1, angiopoietin-like protein 4 (ANGPTL4), and monocyte chemoattractant protein-1 (MCP-1) as adipo-myokines. Today, meteorin-like hormone (Metrnl) [38], glypican-4 (GPC-4) [39], and irisin [40] need to be added to the list. Although the number is increasing, only a limited number of cytokines and chemokines were investigated in terms of lifestyle. Main findings are briefly summarized in Table 2 and Table 3. In terms of myokines, many other cytokines seem to be involved in the crosstalk between skeletal muscle and adipose tissue. In addition to IL-6 and myostatin, IL-15, irisin, and adiponectin seem to have the decisive role in this “conversation” between adipose tissue and skeletal muscle influencing metabolic health (modified By Reference [41]). A general overview of the already more or less known adipo-myokines, myokines, and adipokines is summarized in Table 2 and Table 3.

Surprisingly, no investigation in the current literature explicitly investigated the link between the MHO or MUO phenotypes and myokines. Only one study by Carvalho et al. [84] examining 61 adults aged 20–45 years was able to show a correlation between myostatin levels and TNF-α, and between leptin/adiponectin ratio and VO_2_ peak [84]. In a different investigation, 10 metabolically healthy obese women had higher cardiorespiratory fitness and lower TNF-α levels than 10 age- and weight-matched women with metabolic syndrome [85]. However, these investigations were cross-sectional analyses, as were most other studies that explored adipokines and myokines in this context. In the Copenhagen Aging and Midlife Biobank, Wedell-Neergaard et al. [86] examined cardiorespiratory fitness levels, plasma levels of cytokines, and high-sensitivity C-reactive protein in 1293 participants aged 49–52 years. Fitness was inversely associated with high-sensitivity C-reactive protein, IL-6, and IL-18, and directly associated with the anti-inflammatory cytokine IL-10, but not associated with TNF-α, interferon gamma, or IL-1β. In a parallel study, lower fitness levels were associated with both abdominal adiposity and low-grade inflammation independent of BMI [87]. 

Lee et al. [88] analyzed the association between the adipokines IL-6, MCP-1, TNF-α, and adipocyte fatty acid-binding protein (A-FABP) and metabolic health in 456 subjects (303 men and 153 women; mean age 40.5 years). In a model by Wildman et al. [89], participants were separated into four groups: metabolically healthy or unhealthy and obese or non-obese. Differences in TNF-α levels and A-FABP were found between metabolically healthy subjects and MUNW, but no differences were shown in IL-6 and MCP-1 levels. In light of their results, they called for more research to explore these correlations; unfortunately, neither physical activity/exercise nor cardiorespiratory fitness were taken into account. 

Arsenault et al. [90] investigated the effect of exercise training on inflammatory markers in hypertensive, postmenopausal, metabolically healthy overweight or obese women. The participants (32.0 ± 5.7 kg/m^2^; mean age = 57.3 ± 6.6 years) underwent a six-month exercise intervention program four times per week at 50% VO_2_ max. The training significantly increased cardiorespiratory fitness, but no changes were observed for plasma levels of C-reactive protein, IL-6, TNF-α, and adiponectin. A possible explanation for this could be that participants were considered metabolically healthy per se, and that body composition was not considered because BMI and waist circumference were used as parameters. Training intensity may also have been too low. While we can only speculate about the reasons behind the findings, it does underline how much the chosen methodology and study population may influence study results.

In an investigation by Gómez-Ambrosi et al. [91], the inflammatory potential of 222 MHO and 222 MUO individuals was compared with that of 255 matched normal-weight individuals. Both groups showed an increased cardiometabolic risk and no differences in adipokine levels compared with normal-weight subjects. Consequently, the authors asked for a more precise definition of metabolic health. This issue is certainly relevant for most studies presented in this article. For instance, an analysis of sedentary behavior in the English Longitudinal Study of Aging which counted 4931 participants (mean age 65 years) could not show any differences between the MHO, MUO, and MUNW groups [92].

## 5. Body Composition and Its Influence on Metabolic Health

The previous paragraphs illustrated that exercise and sport are not just means to increase energy consumption, but that the associated development of a high amount of muscle mass elicits the release of myokines, generating an intensive crosstalk between organs. Brown adipose tissue (BAT) and its influence on health may present an important crossover point between fat and muscle mass. In contrast to white adipose tissue (WAT), BAT dissipates lipids in the form of “heat” via β-adrenergic stimulations (e.g., during exercise) or cold exposure. Adipocytes in BAT appear as multilocular cells with small lipid droplets and have a large number of mitochondria and upregulated mitochondrial uncoupling protein 1 (UCP1), which is embedded in the inner membrane of the mitochondrion and uncouples oxidative respiration from ATP synthesis. Whereas classical BAT is observed in specific regions of the body such as the interscapular region and kidney and constitutively sustains its thermogenic activity without any external stimuli, the inducible BAT, known as brite (brown in white), beige, or brown-like adipose tissue, is present within the WAT, and its amount and activity are induced by external stimuli [93]. 

The adipo-myokine irisin is thought to play a central role in the browning processes. First described by Boström et al. [40], irisin is secreted mainly in skeletal muscle, especially in the perimysium, endomysium, and nuclear parts, although adipose tissue, pancreas, sebaceous glands, and cardiac muscle were identified as secretory tissues [94]. Irisin is secreted into the circulation after transcription from its *FNDC5* gene and proteolytic cleavage at the extracellular surface of cells [40]. This is thought to be triggered by the peroxisome proliferator-activated receptor (PPAR) gamma coactivator 1α (PGC-1α) [95]. Exercise stimulates cell signaling pathways that converge on and increase PGC-1α, a well-known activator of the transcription of mitochondrial transcription factor A (TFAM) and mitochondrial biogenesis [96]. The relationship between physical activity or exercise and PGC-1α and its effects on mitochondrial biogenesis and function are not clear [97,98,99]. There are some studies [51,52] which investigated the link between PGC-1α with irisin/fibronectin type III domain-containing protein 5 (FNDC5) in muscle in response to acute exercise and showed an increase in muscle FNDC5 messenger RNA (mRNA), while one study [100] detected a positive association of PGC-1α with FNDC5 in muscle. According to chronic exercise, only two studies [52,101] showed increased PGC-1α and FNDC5 mRNA in muscle, while a predominant number of studies [51,102,103,104] showed no effect of chronic exercise [97,98]. No intervention explored a possible link with MHO. However, Bonfante et al. [105] showed that a higher level of FNDC5/irisin in 20 obese men was associated with better triglyceride levels (*p* = 0.01), lower insulin resistance, risk of type 2 diabetes development, and the tendency to lower serum resistin. Exploring mitochondrial health in this context would exceed the scope of this analysis, and research about metabolic health and sport is sparse. However, a study with 60 participants [106] indicated that MHNW subjects have the most favorable metabolic profiles, while MHO subjects show small alterations, and abnormal diabetic obese individuals have the most unfavorable metabolic profiles [106].

## 6. Early-Life Programming and the Influence of Different Adipokines, Myokines, and Adipo-Myokines 

Today, it is undisputed that early childhood influences metabolic health (see Figure 1). For instance, in the Generation R Study, a population-based prospective cohort investigation among 4871 mothers, fathers, and their children, Gaillard et al. [107] examined the associations of both maternal and paternal pre-pregnancy BMI with childhood body fat distribution and cardiometabolic outcomes six years after birth. Children from obese mothers had a nearly four-fold increased risk of childhood overweight and clustering of cardiometabolic risk factors (odds ratio, 3.00) compared with children from normal-weight women. Furthermore, higher parental pre-pregnancy BMI was associated with higher childhood BMI, total body and abdominal fat mass, systolic blood pressure, and insulin levels, and lower HDL levels. 

Cytokines seem to have a central part in this association. In 2016, we published the so-called adipokine–myokine–hepatokine compartment model [108]. Based on the knowledge at the time, the interrelations between skeletal muscle mass, adipose tissue, and maternal liver, and the questionable role of the placenta were investigated in terms of their influence on metabolic regulation in children and how they may be affected by exercise [108]. Knowledge about cytokine and hormone production and secretion of a specific organ is broad, yet little is known about the complex interplay of cytokines, especially with regard to their activities at rest and during different types of physical stress, and how this might affect metabolic health. Below, we explore different biomarkers that are known to be affected by exercise and physical activity during pregnancy, including leptin, brain-derived neurotrophic factor (BDNF), IL-6, irisin, and myostatin. While most studies examined the effect of exercise on those markers, only a few focused on associations taking cardiorespiratory fitness levels or body composition into account.

In a comparative study, we analyzed the long-term effects of an exercise program during pregnancy on metabolism, weight gain, body composition, and changes in leptin and BDNF [109]. Human and animal models were synchronized according to study design, age, and serum parameters. Regular physical activity led to a 6% lower fat mass, 40% lower leptin levels, and an increase of 50% BDNF levels in humans compared with controls, which was not observed in mice. However, with regard to long-term effects, the offspring of exercising mouse dams had significantly lower fat mass and leptin levels than controls. Furthermore, serum BDNF levels were elevated three-fold in the exercise offspring group compared with control [109]. In summary, this shows that lifestyle factors, especially regular physical activity, can shape the metabolic profile of children in the long term.

Because leptin seems to have far-reaching influences in early childhood, this biomarker is discussed in a little bit more detail. According to Vicker et al. [110], leptin is posited to exert programming effects on central and peripheral energy-regulating pathways during a critical period of fetal and infant development. Accordingly, several researchers [111,112] investigated leptin as a candidate prognostic biomarker for obesity risk in later life. It was documented that umbilical cord blood leptin levels are positively correlated with neonatal body weight and fat mass [113]. Moreover, there is evidence that maternal serum concentration of leptin correlates with pre-pregnancy BMI, weight gain during pregnancy, and cord leptin levels [114].

In a follow-up of a cross-sectional study with 76 mother–child pairs, our research group [111] examined the effects of maternal anthropometric, sociodemographic, and lifestyle factors on maternal and cord-blood leptin levels at birth, and on the development of BMI standard deviation scores (SDS) in offspring up to one year of age. We demonstrated that higher maternal and lower cord-blood leptin levels are associated with a higher BMI SDS increase during the first year of life. Maternal leptin is influenced by maternal BMI and weight gain during pregnancy, and cord-blood leptin is influenced by maternal physical activity [111]. These results are in line with findings by Simpson et al. [115], who found that higher cord-blood leptin was associated with higher *z*-scores of fat mass, waist circumference, and BMI at nine years of age, although they did not take lifestyle factors such as physical activity into account.

The abovementioned myokine BDNF is necessary for placental development, fetal growth, glucose metabolism, and energy homeostasis. It has functions in cognition and neuroplasticity in the hypothalamus and has an influence on appetite regulation and insulin sensitivity in peripheral tissues. Although it is still unknown where exactly BDNF is produced peripherally, BDNF levels in the brain seem to correlate with serum BDNF concentrations [116]. BDNF crosses the blood–brain barrier in a bidirectional manner [117]. Lommatzsch et al. [118] suggested that blood levels of BDNF may reflect brain levels and vice versa. Moreover, we demonstrated in a previous human study that umbilical-cord BDNF is correlated with maternal BDNF levels [119]. In human studies, there is no evidence to date as to how changes in the mother’s BDNF values have a long-term effect on the metabolic pattern in the offspring. Camargos et al. [120] evaluated adipokines, cortisol, BDNF, and redox status in 25 overweight/obese infants versus 25 normal-weight peers between six and 24 months of age. Overweight or obese infants presented higher levels of leptin, adiponectin, BDNF, and cortisol and lower levels of thiobarbituric acid-reactive substances (TBARS), as well as catalase and superoxide dismutase activity, than their normal-weight peers. The authors stated that these results indicate neuroendocrine inflammatory response changes in overweight/obese infants. Regarding the effects of physical activity on BDNF levels in children, Walsh et al. [121] examined the associations between changes in diabetes risk factors and changes in BDNF levels after six months of exercise training (aerobic and/or resistance training) in 202 14–18-year-old adolescents with obesity. In this study, exercise-induced reductions in some diabetes risk factors (most notably fasting glucose and beta cell insulin secretory capacity) were associated with increases in BDNF in adolescents with obesity. The authors suggested that exercise training may be an effective strategy to promote metabolic health and increase BDNF. These findings are in line with the results of Mora-Gonzalez et al. [122]; evaluating the effect of physical activity on BDNF levels in 97 overweight or obese children aged eight to 11 years, they found that physical activity was positively correlated with BDNF levels. Although there is currently no evidence about the long-term effects of physical activity during pregnancy on BDNF levels in children, based on the presented results and animal experimental data [123,124], it can be postulated that the metabolic and neurodevelopment profile of children can be shaped in some way.

IL-6 appears to be another important biomarker in early childhood. Our cooperating working group [125] aimed to identify molecular mechanisms of cytokines in the offspring who are affected by maternal exercise during pregnancy. The authors found an almost four-fold increase in serum IL-6 with a clear activation of Janus kinase/signal transducer and activator of transcription signaling in the WAT and hypothalamus of obese offspring, which was completely blunted by maternal exercise during pregnancy. The altered hypothalamic global gene expression in obese offspring showed partial normalization in the obese running offspring group, especially with respect to IL-6 action [125].

In a human-based study of 124 children aged 10.0 ± 0.9 years, Hosick et al. [126] compared markers of inflammation (IL-6, TNF-α) between normal-weight youth of high or low aerobic fitness to obese youth of high or low fitness. They found that higher levels of VO_2_ max are associated with lower levels of IL-6, independent of obesity. 

Furthermore, irisin may play a crucial role in the interplay between adipokines and myokines during early childhood. It is known that the level of irisin in pregnant women is higher than that of non-pregnant women. Irisin may also contribute to the physiological insulin resistance found in pregnancy [127] and it has an important role in controlling maternal and fetal glucose homeostasis. Low irisin cord-blood levels are associated with newborn growth delay [128] and low proportions of brown fat tissue. Ökdemir et al. [129] aimed to investigate the relationships between irisin, insulin, and leptin levels and maternal weight gain, as well as anthropometric measurements in the newborn. Eighty-four mothers with a mean age of 29.8 ± 5.2 years and their newborns were enrolled in the study. The authors found a negative correlation between the anthropometric measurements of the “appropriate for gestational age” newborns and irisin levels. This correlation was not observed in “small for gestational age” and “large for gestational age” babies. To our knowledge, there are no studies that examined the associations between irisin levels in pregnancy and the development of children in later life. In addition, the association between physical activity during pregnancy and irisin concentrations in the offspring remains to be investigated. Irisin recently became a focus of research in the field of pediatric obesity, because it might play an important pathophysiological role in metabolic dysfunctions and its complications. The positive correlations between leptin levels and anthropometric and metabolic parameters in children with obesity and metabolic syndrome are well known [130,131]. In a cross-sample of 126 Mexican children aged 6–12 years, Gonzales-Gil et al. [132] characterized the association between irisin and adipokines, as well as the cardiometabolic risk factors and anthropometric parameters in children with obesity or metabolic syndrome and in normal-weight children. 

They demonstrated that irisin plasma levels were negatively correlated with leptin levels. Furthermore, irisin levels were significantly lower in the obese and metabolic syndrome groups than in the normal-weight group. Stepwise multiple linear regression analysis was conducted to determine whether body composition (lean-fat ratio), metabolic parameters (triglyceride, HDL, and glucose levels), and physical activity in hours per week influenced irisin levels. The lean–fat ratio was found to be the only significant determinant of irisin levels with the model explaining 22.7% of the variance in irisin levels.

Myostatin is secreted during embryonic development, and its function is to limit muscle growth physiologically during development [41]. In the human placenta, the expression of myostatin is negatively correlated with gestational age, and, in placental explants, myostatin acts to facilitate glucose uptake [133]. Myostatin expression is known to be higher in the placenta of pregnancies complicated by preeclampsia [133]. Moreover, Peiris et al. [134] found complications in myostatin protein expression in human placenta from women with gestational diabetes mellitus compared with normal glucose tolerant pregnancies. Compared with lean women, the placentas of obese normal glucose-tolerant women were lower in myostatin dimer expression [134]. The authors concluded that myostatin expression in placental tissue is altered under stress conditions (e.g., obesity and abnormal glucose metabolism) found in pregnancies complicated by gestational diabetes mellitus. In a longitudinal study with 125 infants, De Zegher et al. [135] tested whether large-born infants from non-diabetic mothers develop an early surplus of lean mass while having a lower myostatinemia. They confirmed that breast-fed large-for-gestational-age infants from non-diabetic mothers developed a marked surplus of lean mass at four months of age while maintaining low levels of circulating myostatin. The authors concluded that the fetal–neonatal control of myostatinemia deserves further attention, because it might become a target of interventions aiming to reduce the risk for diabetes in later life by augmenting myogenesis in early life [135]. No studies were conducted to date to investigate the effect of regular physical activity during pregnancy on myostatin in both mother and child. Based on the results of Peiris et al. [134], it might be postulated that placental myostatin could affect glucose homoeostasis and/or cytokine production, which in turn might be influenced by exercise. 

## 7. Discussion

In addition to obesity, physical inactivity/sedentary behavior is a global health problem, and recent data indicate that approximately one-third of the world’s adult population is physically inactive. This means that these individuals do not perform the minimum of 150 min per week of moderate-to-vigorous aerobic physical activity recommended by the WHO [27,136]. Importantly, physical inactivity is directly related to higher risk rates for the majority of NCDs. The purpose of this review was to illustrate the association between exercise/physical activity or cardiorespiratory fitness and metabolic health with regard to myokines, adipokines, and adipo-myokines, as well as the influence of early-life factors. Although further research is needed to specifically recommend the type of exercise to be used, as well as corresponding duration, volume, and intensity that would be required to elicit the desired effects, the benefit of exercise in the context of obesity and NCDs remains undisputed. It seems critical that an (exercise) stimulus needs to pass a certain threshold level to be effective [99], but this assumption may be based on a methodological problem. For instance, blood parameters might need to reach a certain level to detect significant differences, although this does not necessarily imply a treatment effect. From a public health perspective, it seems most important to simply “get started”, especially because people with a low fitness level will see the greatest health benefits from taking up exercise, particularly in the beginning [10,137]. Regularly performing moderate-to-vigorous intensity, endurance-based activities, for example, walking, jogging, or cycling, leads to an increase of ~10% in “fitness” in different populations (e.g., those who are healthy or obese, or who have coronary artery disease or diabetes) or different obesity phenotypes (e.g., MUO, MHO), which highlights how the majority of adults can acquire clinically important gains in cardiorespiratory fitness. It may not be possible to find an answer as to what is the “right” type of exercise because of the great complexity of the topic. Myokines, for example, were studied for 20 years; many new mechanisms were discovered, some of which we tried to cover in this paper, yet the analysis shows just how many questions are still left unanswered. 

There are several limitations to our article. This is a narrative review in which we attempted to explore the interrelations between exercise and, in particular, cardiorespiratory fitness, metabolic health, and obesity, taking into consideration underlying mechanisms and developments in early childhood. We did not perform a systematic literature review about the influence of different types of exercise or training methods. It should be taken into account that the presented results, for example, irisin/FNDC5, may be affected by different subjects’ age, conditioning status, and exercise intensity [97,98]. Moreover, different analytical measurements might lead to different results, which were not taken into account. Studies that were included were mostly performed with adult participants. We focused on human interventions and only integrated animal studies to elucidate a specific aspect in more detail. The analysis of adipokines, myokines, and adipo-myokines mentioned herein does not claim to be exhaustive. For instance, epigenetic aspects and/or the role of hepatokines were not taken into consideration. The demand for one uniform definition of metabolically healthy or unhealthy obese individuals (MHO/MUO), or even of metabolic health itself, reflects the difficulties and basic issues involved in this topic and highlights the need for a general consensus. This consensus should consider cardiorespiratory or muscular fitness and should subsequently be tested in future investigations. Equally, interventions should be designed in a manner that allows for an appropriate comparison to make final assumptions. It can only be speculated as to what extent novel techniques such as the secretomic technique can help unravel the underlying mechanisms and their complex interrelations. Mitochondrial biogenesis seems to be one of the central aspects in this issue. Already in 1967, it was shown that physical activity leads to an increased number and improved function of mitochondria [138]; over 50 years later, this issue seems to be more relevant than ever before in light of the rising prevalence of obesity and the adverse changes in body composition toward an unfavorable ratio of fat to muscle mass. As such, there is now an increased focus on sarcopenia, which is usually mentioned in the context of aging and chronic disease [139]. In light of the imminent cost explosion that is to be expected due to the far-reaching consequences of these health issues, it is crucial to understand which patient groups require more or less intensive treatment concepts and prevention. From a practical point of view, it seems sensible to choose an approach that first and foremost focuses on individual tendencies and individual health status to get and keep people physically active. Finally, a political dimension is added to this discussion. Especially in terms of epigenetic aspects, it is crucial to create active living spaces to provide the groundwork for healthy aging. Findings from basic scientific research can help explain and clarify theses connections and their meanings, and facilitate resolving them on a political level. 

## 8. Conclusions

At present, a great body of research on metabolic health and different phenotypes of obesity (MHO/MONW/MUO) exists. However, several questions remain unanswered. Despite the knowledge that different “obesities” exist, no refined metabolic health definition was developed. A better understanding of both the (early-life) lifestyle factors and the underlying mechanisms that mediate different phenotypes is necessary in terms of the tailored prevention and personalized treatment of obesity. 

## Figures and Tables

**Table 1:** German physical activity recommendations for adults according to Reference [27].

German Physical Activity Recommendations for Adults
Adults should be physically active on a regular basis, which can help to achieve significant health effects and to reduce the risk of developing chronic diseases.
The greatest health benefits take place when individuals who were entirely physically inactive become somewhat more active; this means that all additional physical activity is linked to health benefits and that every single step away from physical inactivity is important, no matter how small, and promotes health.
To maintain and promote health comprehensively, the following minimum recommendations apply: – adults should have moderate-intensity aerobic physical activity for at least 150 minutes/week, where possible (e.g., 5 × 30 minutes/week); or – at least 75 minutes/week of vigorous-intensity aerobic physical activity; or – aerobic physical activity in a corresponding combination of both intensities; and – should group the overall activity in at least 10-min individual units distributed over days and weeks (e.g., at least 3 × 10 minutes/day on five days per week).
Adults should also have muscle-strengthening physical activity at least two days per week.
Adults should avoid long and uninterrupted sitting times and should regularly interrupt sitting with physical activity, where possible.
Adults can achieve further health effects if they increase the volume and/or intensity of physical activity above the minimum recommendations.

**Table 2:** Adipo-myokines, myokines, and adipokines in different tissues, according to References [13,14,37,38,39,40,41,42,43,44,45,46,47,48].

Name	Effects—Skeletal Muscle	Effects—Adipose Tissue
Adipo-Myokines		
Interleukin-6 (IL-6)	Induces muscle hypertrophy, glucose uptake, glycogen breakdown, and lipolysis; anti-inflammatory effect	Increases lipolysis and free fatty acid (FFA) oxidation in adipocyte, induces adipocyte browning; pro-inflammatory effect
Irisin/fibronectin type III domain-containing protein 5 (FNDC5)	Stimulates glucose uptake and lipid metabolism; involved in muscle growth	Induces adipocyte browning and lipolysis, stimulates glycogenesis, and reduces gluconeogenesis/lipogenesis in liver
IL-15	Stimulates muscle growth and glucose uptake, enhances mitochondrial activity, and exerts anti-oxidative effect	Inhibits lipid accumulation in adipose tissue through adiponectin stimulation
β-aminoiso-butyric acid (BAIBA)	Increases mitochondrial FFA oxidation and ameliorates insulin signaling; anti-inflammatory effect	Increases mitochondria FFA oxidation and browning in adipocytes; reduces hepatic de novo lipogenesis and hepatic endoplasmic reticulum stress
Meteorin-like hormone (Metrnl)	Causes an increase in whole-body energy expenditure; improves glucose tolerance in obese/diabetic mice	Induces adipocyte browning indirectly through regulation of eosinophils
Leukemia inhibitory factor (LIF)	Induces muscle hypertrophy, satellite cell proliferation, regeneration after muscle damage, and glucose uptake	Inhibits adipocyte differentiation
Myostatin	Inhibits muscle hypertrophy	Inhibits myostatin results in adipocyte lipolysis and mitochondrial lipid oxidation; accelerates osteoclast formation
IL-7	Regulates muscle cell development, increases migration of satellite cells	Unknown
Myokines (main effects)		
Fibroblast growth factor 21 (FGF21)	Insulin-responsive myokine involved in the control of glucose homoeostasis, insulin sensitivity, and ketogenesis	Thermogenesis and fat browning in brown (BAT) and white adipose tissue (WAT); increases expression of mitochondrial uncoupling protein 1 (UCP1) and other thermogenic genes in response to cold exposure and β-adrenergic stimulation in both fat depots
Myogenin	Transcription factor; involved in muscle development, myogenesis, and repair	Unknown
Myonectin	Regulates whole-body fatty-acid metabolism	Links skeletal muscle to lipid metabolism adipose tissue and liver
Brain-derived neurotrophic factor (BDNF)	Increases fat oxidation in a 5’ AMP-activated protein kinase (AMPK) -dependent fashion	Size of adipose tissue
Monocyte chemoattractant protein-1 (MCP-1)	Recruitment of monocytes and T lymphocytes; impairs insulin signaling	Involved in low-grade inflammation
Follistatin-like 1 (FSTL1)	Affects glucose metabolism	Correlates with body mass; cardioprotective; improves endothelial function
Angiopoietin-like protein 4 (ANGPTL4)	Increase in FFA	Unknown
Adipokines (main effects)		
Visfatin	Involved in glucose metabolism?	Reduced by exercise?
Resistin	Unknown	Correlates with body fat mass and waist circumference; may cause endothelial dysfunction
Leptin	Increases muscle mass by increasing myocyte cell proliferation and reducing the expression of negative regulators of muscle growth including myostatin, dystrophin, or atrophy markers muscle atrophy F-box (MAFbx) or muscle RING finger 1 (MuRF1); upregulates FNDC5 expression and enhances irisin-induced myocyte proliferation, as well as the muscle growth enhancers myogenin and myonectin; post-exercise decrease	Regulation of energy homeostasis; increases energy expenditure through the stimulation of sympathetic nerve activity in BAT
Adiponectin	Increase fatty-acid oxidation and glucose uptake	Inhibits gluconeogenesis in liver; cardioprotective; increases insulin sensitivity
Tumor necrosis factor alpha (TNF-α)	Reduced after training; increased after very intensive exercise in response to muscle damage; reduced by chronic exercise	Correlates with body fat mass

**Table 3:** Effect of physical activity in human studies (subject age range 18–65 years).

Name	Effects of Physical Activity
Adipo-Myokines	
IL-6	Plasma concentration of IL-6 increases during muscular exercise. The combination of mode, intensity, and duration of the exercise determines the magnitude of the exercise-induced increase of plasma IL-6 [42]. IL-6 levels were 13.2-fold increased directly after a 35-km long-distance trail run [49].
Irisin/FNDC5	Controversially discussed: Two-fold increase of circulating irisin after 10 weeks of endurance training [40] vs. no increase in irisin after 8 weeks of intermittent sprint running or after 21 weeks of combined endurance and strength training [50,51]. Reduction of circulating irisin in response to 12 weeks of combined endurance and strength training [52] vs. an increase acutely (~1.2-fold) just after acute exercise [52].
IL-15	Controversially discussed: Strength/resistance training leads to an increase in IL-15 messenger RNA (mRNA) level in skeletal muscles dominated by type 2 fibers [53,54]. Short bout of endurance exercise also increases levels of IL-15 in lean subjects, as well as in overweight/obese, subjects [55]. A 2.22-fold increase in serum IL-15 levels following an acute long-distance trail run was also found by Yarcic et al. [49] In contrast, neither sprint interval training (SIT) or combined aerobic and resistance training (A + R) altered IL-15 measured 48 h after exercise in overweight type 2 diabetes (T2D) [56].
BAIBA	Acute aerobic exercise induces a 13% and 20% increase in R-BAIBA and S-BAIBA, respectively [57]. A chronic elevation of 17% was also observed following 20 weeks (3 days/week) of aerobic exercise in previously sedentary and healthy subjects [58].
Metrnl	Lack of data in human studies: Aerobic exercise/swimming (40 min on three non-consecutive days) in temperate (24–25 °C), warm (36.5–37.5 °C), and cold (16.5–17.5 °C) water leads to an increase after exercise in temperate and warm water and a significant decrease in cold water in overweight women [59].
LIF	Aerobic exercise and concentric muscle contractions regulate muscular LIF mRNA expression in humans and lead to an induced expression of LIF in human skeletal muscle [60]. This was also confirmed for resistance exercise [48,61].
Myostatin	Myostatin mRNA expression was reduced in skeletal muscle after acute and long-term exercise and was even further downregulated by acute exercise on top of 12-week training in previously sedentary men [62]. 15 units of a high-intensity circuit training (HICT) program (3×/week for 5 weeks) with own body weight induced the drop of myostatin concentration but significantly only among middle-aged women [63].
IL-7	Lack of data in human studies: Cyclists show higher serum levels of IL-7 compared to less active counterparts [64].
Myokines (main effects)	
FGF21	Increase in serum FGF21 levels in runners after 2 weeks of training [65] and after an acute session of running exercise [66].
Myogenin	Myogenin increases after eccentric resistance training [67,68].
Myonectin	Controversially discussed: Aerobic moderate-intensity exercise leads to a significant reduction in the amount of myonectin in older and younger patients [69]. In contrast, Seldin et al. [44] and Poranjibar et al. [70] found an increase in myonectin expression in muscle and circulation.
BDNF	Increase in BDNF concentrations after aerobic exercise is associated with the amount of aerobic energy required by exercise in a dose-dependent manner [71]. High-intensity and high-volume resistance training lead to elevations in BDNF concentrations [72].
MCP-1	Lack of data in human studies: Low-intensity exercise (walking 10,000 steps/day, 3×/week for 8 weeks) downregulates MCP-1 [73].
FSTL1	Acute sprint interval exercise, as well as acute aerobic exercise, increases FSTL1 [74,75].
ANGPTL4	Controversially discussed: Increase in the gene expression of ANGPTL4 after 4 and 8 h following muscle contraction stimulated in myocytes during exercise using electrical pulse stimulus [76] vs. downregulation of ANGPTL4 in the exercised leg after acute endurance exercise [77].
Adipokines (main effects)	
Visfatin	Lack of data in human studies: Circuit resistance training (3×/week with intensity at 55% of one-repetition maximum) for 8 weeks reduces levels of visfatin [78].
Resistin	Anaerobic exercise might decrease levels of resistin [79]. Resistance training leads to a decrease in resistin after 24 and 48 h compared with baseline and a decline in baseline and immediately after levels compared with pre-training [80].
Leptin	Aerobic exercise leads to lower leptin levels in different population groups (prediabetic/diabetic adults; overweight/obese adults; different age and sex) [81,82,83].
Adiponectin	Aerobic exercise leads to an increase of adiponectin levels in different population groups (prediabetic/diabetic adults; overweight/obese individuals) [81,82].
TNF-α	Only highly strenuous, prolonged exercise such as marathon running results in a small increase in the plasma concentration of TNF-α [42]. The serum level of TNF-α was significantly downregulated after eccentric resistance exercise in non-athletes [54].

## Abbreviations

WHO	World Health Organization
NCDs	Non-Communicable Diseases
BMI	Body Mass Index
MONW	Metabolically Obese, Normal Weight
MUO	Metabolically Unhealth Obese
MHO	Metabolically Healthy Obese
IL	Interleukin
RR	Risk Ratio
METs	Metabolic Units
NHANES	National Health and Nutrition Examination Survey
CVD	Cardiovascular Disease
NCEP APT III	National Cholesterol Education Program Adult Treatment Panel III
FSTL 1	Follistatin-like 1
ANGPTL4	Angiopoietin-like protein 4
MCP-1	Monocyte Chemoattractant Protein-1
Metrnl	Meteorin-like hormone
GPC-4	Glypican-4
TNF-α	Tumor Necrosis Factor alpha
A-FABP	Adipocyte Fatty Acid-Binding Protein
BAT	Brown Adipose Tissue
WAT	White Adipose Tissue
UCP1	Uncoupling protein 1
FNDC5	Fibronectin type III domain-containing protein 5
PPAR gamma	Peroxisome proliferator-activated receptor gamma
PGC-1 alpha	Peroxisome proliferator-activated receptor-gamma coactivator-1 alpha
TFAM	Mitochondrial Transcription Factor A
BDNF	Brain-Derived Neurotrophic Factor
SDS	Standard Deviation Scores
TBARS	Thiobarbituric Acid-Reactive Substances
Jak	Janus kinase
STAT	Signal Transducer and Activator of Transcription
FFA	Free Fatty Acid
MuRF1	muscle RING finger 1
MAFbx	muscle atrophy F-box
AMPK	5′ AMP-activated protein kinase
